# Multidisciplinary Tinnitus Research: Challenges and Future Directions From the Perspective of Early Stage Researchers

**DOI:** 10.3389/fnagi.2021.647285

**Published:** 2021-06-11

**Authors:** Jorge Piano Simoes, Elza Daoud, Maryam Shabbir, Sana Amanat, Kelly Assouly, Roshni Biswas, Chiara Casolani, Albi Dode, Falco Enzler, Laure Jacquemin, Mie Joergensen, Tori Kok, Nuwan Liyanage, Matheus Lourenco, Punitkumar Makani, Muntazir Mehdi, Anissa L. Ramadhani, Constanze Riha, Jose Lopez Santacruz, Axel Schiller, Stefan Schoisswohl, Natalia Trpchevska, Eleni Genitsaridi

**Affiliations:** ^1^Department of Psychiatry and Psychotherapy, University of Regensburg, Regensburg, Germany; ^2^Centre National de la Recherche Scientifique, Aix-Marseille University, Marseille, France; ^3^Hearing Sciences, Mental Health and Clinical Neurosciences, School of Medicine, University of Nottingham, Nottingham, United Kingdom; ^4^Otology & Neurotology Group CTS 495, Department of Genomic Medicine, GENYO - Centre for Genomics and Oncological Research Pfizer/University of Granada/Junta de Andalucía, PTS, Granada, Spain; ^5^Department of Otorhinolaryngology and Head & Neck Surgery, University Medical Center Utrecht, Utrecht, Netherlands; ^6^Department of Clinical and Experimental Neuroscience, University Medical Center Utrecht Brain Center, Utrecht University, Utrecht, Netherlands; ^7^Cochlear Technology Centre, Mechelen, Belgium; ^8^Laboratory of Lifestyle Epidemiology, Department of Environmental Health Sciences, Istituto di Ricerche Farmacologiche Mario Negri IRCCS, Milan, Italy; ^9^Hearing Systems, Department of Health Technology, Technical University of Denmark, Lyngby, Denmark; ^10^Oticon A/S, Smoerum, Denmark; ^11^Interacoustics Research Unit, Lyngby, Denmark; ^12^Institute of Databases and Information Systems, Ulm University, Ulm, Germany; ^13^Department of Otorhinolaryngology Head and Neck Surgery, Antwerp University Hospital, Edegem, Belgium; ^14^Department of Translational Neurosciences, Faculty of Medicine and Health Sciences, Antwerp University, Wilrijk, Belgium; ^15^WS Audiology, Lynge, Denmark; ^16^Ear Institute, University College London, London, United Kingdom; ^17^University of Zurich, Zurich, Switzerland; ^18^Department of Otorhinolaryngology, Head and Neck Surgery, University Hospital Zurich, Zurich, Switzerland; ^19^Experimental Health Psychology, Faculty of Psychology and Neuroscience, Maastricht University, Maastricht, Netherlands; ^20^Health Psychology Research Group, Faculty of Psychology and Educational Sciences, University of Leuven, Leuven, Belgium; ^21^Department of Otorhinolaryngology, Head and Neck Surgery, University of Groningen, University Medical Center Groningen, Groningen, Netherlands; ^22^Graduate School of Medical Sciences (Research School of Behavioral and Cognitive Neurosciences), University of Groningen, Groningen, Netherlands; ^23^Institute of Distributed Systems, Ulm University, Ulm, Germany; ^24^Radiological Sciences, Mental Health and Clinical Neurosciences, School of Medicine, University of Nottingham, Nottingham, United Kingdom; ^25^Sir Peter Mansfield Imaging Centre, School of Medicine, University of Nottingham, Nottingham, United Kingdom; ^26^Chair of Neuropsychology, Department of Psychology, University of Zurich, Zurich, Switzerland; ^27^Department of Physiology and Pharmacology, Experimental Audiology Laboratory, Karolinska Institutet, Stockholm, Sweden; ^28^Nottingham Biomedical Research Centre, National Institute for Health Research, Nottingham, United Kingdom

**Keywords:** tinnitus, review, heterogeneity, standardization, interdisciplinary collaborations, big data, treatment development

## Abstract

Tinnitus can be a burdensome condition on both individual and societal levels. Many aspects of this condition remain elusive, including its underlying mechanisms, ultimately hindering the development of a cure. Interdisciplinary approaches are required to overcome long-established research challenges. This review summarizes current knowledge in various tinnitus-relevant research fields including tinnitus generating mechanisms, heterogeneity, epidemiology, assessment, and treatment development, in an effort to highlight the main challenges and provide suggestions for future research to overcome them. Four common themes across different areas were identified as future research direction: (1) Further establishment of multicenter and multidisciplinary collaborations; (2) Systematic reviews and syntheses of existing knowledge; (3) Standardization of research methods including tinnitus assessment, data acquisition, and data analysis protocols; (4) The design of studies with large sample sizes and the creation of large tinnitus-specific databases that would allow in-depth exploration of tinnitus heterogeneity.

## 1. Introduction

Tinnitus, the perception of sound in the absence of an external acoustic stimulus, is a common condition with prevalence often estimated between 10 and 15% in the adult population (Baguley et al., 2013). The burden of tinnitus on society is not only reflected in its impact on an individual's Quality of Life (QoL), but also on that of their significant other (Kennedy et al., 2004; Zeman et al., 2014; Weidt et al., 2016; Hall et al., 2018a). Tinnitus management requires a multidisciplinary approach due to the heterogeneity of tinnitus and the absence of a cure (Cima et al., 2020). The average cost of tinnitus treatment per patient per year is approximately USD 660 in the United States (USA) (Goldstein et al., 2015), GBP 717 in the United Kingdom (UK) (Stockdale et al., 2017), and EUR 1,544 in the Netherlands (Maes et al., 2013).

The tinnitus research field has benefited from multidisciplinary consortia such as the Tinnitus Research Initiative (TRI) (Langguth et al., 2006), the TINnitus research NETwork (TINNET, 2014), the European School on Interdisciplinary Tinnitus Research (ESIT) (Schlee et al., 2018), the Tinnitus Genetic and Environmental Risks (TIGER, 2019), the TINnitus - Assessment, Causes and Treatments (TIN-ACT, 2017), and the Unification of Treatments and Interventions for Tinnitus Patients (Schlee et al., 2021). Despite all the efforts by these welcomed initiatives, tinnitus still remains an under-researched field. Although the annual number of scientific publications on other conditions such as depression, anxiety, and deafness has increased drastically in the last years, this has not been the case for tinnitus (McFerran et al., 2019).

Many long-established research questions still remain unanswered, including the underlying pathophysiology, and whether there can be a cure (McFerran et al., 2019). However, there are many challenges when investigating these questions, such as the lack of standardization in tinnitus research, the lack of objective measures in the assessment of tinnitus, and the under-characterized heterogeneous nature of tinnitus (Langguth et al., 2011a; Jackson et al., 2019). In this review, the various challenges hindering progress in tinnitus research are discussed, and suggestions for future research priorities are provided with a focus on interdisciplinary approaches. These suggestions were based on the experiences and expertise of young researchers affiliated with two of the biggest training school consortia for tinnitus, ESIT and TIN-ACT. No formal search for the identification of relevant references was conducted, but literature databases were searched in a non-systematic way. This article is divided into dedicated sections for the main categories of tinnitus research, each providing an overview of the current knowledge, a summary of the most significant challenges, and suggestions for essential next steps.

## 2. Tinnitus Heterogeneity

### 2.1. Overview

Tinnitus is characterized by heterogeneous manifestations and underlying mechanisms (Cederroth et al., 2019a). Great effort has been made to understand this heterogeneity and identify more homogeneous subgroups of the investigated underlying conditions, or the dimensions across which to characterize individual cases. Measuring heterogeneity, however, is a big challenge (Nunes et al., 2020), and identifying subtypes is an open question for numerous conditions including mental disorders, diabetes, and asthma (Ahlqvist et al., 2018; Brückl et al., 2020; Schoettler and Strek, 2020). In the case of tinnitus, efforts to understand heterogeneity are still ongoing (Cederroth et al., 2019a). In the following sections, commonly investigated aspects of tinnitus heterogeneity are discussed, and important challenges and future directions are highlighted.

One aspect of tinnitus heterogeneity is with regards to its pathophysiology. It is well-known that the two main types of tinnitus, subjective and objective, are generated by different underlying mechanisms (Zenner, 1998; Lockwood et al., 2002). Subjective tinnitus refers to a phantom perception, whereas objective tinnitus refers to perceived sounds generated from an acoustic source located within the body. Pathologies causing objective tinnitus are rather heterogeneous and research to understand and treat this form of tinnitus is ongoing (Liu et al., 2019; Choi et al., 2020). It is, however, far less common than subjective tinnitus and its underlying mechanisms are usually clearer (Tunkel et al., 2014). Therefore, the majority of tinnitus research focuses on subjective tinnitus, which is also the focus of this article (henceforth referred to as tinnitus).

Current evidence suggests that the pathophysiology of (subjective) tinnitus is also multifactorial (Vanneste and De Ridder, 2016; Schmidt et al., 2017). Such heterogeneity may be a major contributing factor for the limited progress in understanding the neural correlate of tinnitus and the inexistence of effective treatments (Landgrebe et al., 2010; Cederroth et al., 2019a; Kleinjung and Langguth, 2020). Although various underlying pathologies (e.g., different pathologies affecting hearing such as in presbycusis) can be associated with tinnitus (Baguley et al., 2013), whether or not a common pathophysiological pathway for tinnitus emergence exists remains unanswered.

Due to the lack of an objective measure and the limited understanding of the mechanisms, research on tinnitus heterogeneity and tinnitus subtypes most often focuses on phenotypic heterogeneity of people with tinnitus; that is measurable traits that vary across individuals with tinnitus. These traits can include both general individual characteristics (not specific to people with tinnitus) such as demographics (e.g., gender) and co-existing conditions (e.g., hearing problems), and tinnitus-specific characteristics such as related to the tinnitus perception or its impact on the affected individual (Langguth et al., 2007; van den Berge et al., 2017; Genitsaridi et al., 2020; Van der Wal et al., 2020).

### 2.2. Multiple Aspects of Phenotypic Heterogeneity

#### 2.2.1. Tinnitus-Specific Characteristics

Tinnitus severity, or more broadly, the impact of tinnitus, is arguably the most significant dimension of phenotypic tinnitus heterogeneity. From a clinical perspective, not all cases require an intervention as habituation to the perceived sound can allow an individual to live an unaffected life (Husain, 2016). Heterogeneity of tinnitus severity can be assessed not only by the degree of severity, but also by the domains of life that are affected by tinnitus (Hall et al., 2018a). Many studies have investigated associations between tinnitus severity and a number of other phenotypic characteristics such as presence of comorbidities and personality traits (Hoekstra et al., 2014; Van der Wal et al., 2020), but it remains unclear why some people are distressed by tinnitus while others are not.

Temporal characteristics of tinnitus, particularly tinnitus duration, also contribute to its phenotypic heterogeneity. Most commonly, research in the field focuses on chronic cases, partly because of how difficult it is to recruit individuals with acute tinnitus. Chronic tinnitus can be defined as tinnitus lasting for at least six months (Tunkel et al., 2014), however, there is no universal consensus on the definition (Ogawa et al., 2020).

#### 2.2.2. General Individual Characteristics

In addition, tinnitus heterogeneity can be viewed in terms of coexisting conditions, such as hearing loss and depression, creating a complex, multifaceted clinical picture (Baguley et al., 2013). Cianfrone et al. (2015) proposed a classification system based on the presence or lack of auditory alterations, somatosensory-auditory interactions, and psychopathological disorders, thus highlighting the significance of these comorbidities. The following sections discuss these common tinnitus comorbidities.

Tinnitus is often associated with deficits in auditory function. The majority of tinnitus cases are associated with some degree of hearing loss (67.7% according to Nondahl et al., 2002). However, the mechanisms behind how tinnitus and hearing loss are related still remain unclear. Evidence suggests that subtypes of tinnitus might be associated with distinct profiles of hearing loss, but this should be established in future studies (Vanneste and De Ridder, 2016; Langguth et al., 2017). Importantly, the definition of hearing loss itself is rather ambiguous. The presence or absence of hearing loss is usually clinically determined through pure tone audiometry (PTA) at octave frequencies between 125 and 8,000 Hz. Nevertheless, it is possible that the hearing loss exists at extended high frequencies or between two tested frequencies (Vielsmeier et al., 2015; Lefeuvre et al., 2019; Xiong et al., 2019), despite normal hearing in the range 125–8,000 Hz. Additionally, since audiometry only assesses sensitivity, it does not capture deficits in supra thresholds processing. In the case of cochlear synaptopathy, the hypothesis is that, even though the hearing thresholds appear normal, a loss of cochlear nerve synapses causes hearing deficits, and possibly tinnitus (Schaette and McAlpine, 2011). Such cases of auditory deficits not captured by standard audiological assessment are often referred to as “hidden hearing loss” (Kohrman et al., 2020).

Another hearing-related condition commonly co-existing with tinnitus is hyperacusis. Hyperacusis can be defined as an increased auditory sensitivity, where everyday sounds are perceived as louder or more intense compared to what a person with “normal” hearing function would experience (Fackrell et al., 2017). It is estimated that 40% of patients with tinnitus as a primary complaint also report hyperacusis (Jastreboff and Jastreboff, 2000), while 86% of patients with hyperacusis as a primary complaint also report tinnitus (Anari et al., 1999). However, these estimates should be interpreted cautiously as there is no universally accepted definition of hyperacusis (Tyler et al., 2014c; Aazh and Moore, 2017; Fackrell et al., 2017). As such, definitions and tools used to diagnose hyperacusis vary across studies (Fackrell et al., 2017) and currently, there is no gold standard for hyperacusis diagnosis (Aazh and Moore, 2017). Furthermore, hyperacusis is also considered to be a rather heterogeneous condition. For instance, it has been suggested to differentiate between types of hyperacusis such as loudness, fear, annoyance, and pain (Tyler et al., 2014c). It is therefore essential for studies to provide a clear definition of hyperacusis, and to specify what diagnostic methods were used. Regarding the mechanisms underlying the observed association between tinnitus and hyperacusis, it is speculated that the two conditions might share some common pathophysiological pathways, for example, an increase of auditory central gain (Noreña and Chery-Croze, 2007) and/or disruption of the tensor tympani middle ear muscle after an acoustic shock (Noreña et al., 2018).

Associations between somatosensory system related conditions and tinnitus have been observed for many decades. Indeed, a number of researchers agree that somatosensory system involvement should be considered as an important domain for tinnitus subtyping (Sanchez and Rocha, 2011; Cianfrone et al., 2015; Levine and Oron, 2015; Michiels et al., 2018), with evidence suggesting that the fusiform cells of the dorsal cochlear nucleus play an important role, integrating auditory and somatosensory inputs (Levine, 1999; Shore et al., 2007, 2016; Lanting et al., 2010). Due to the diversity of the criteria used to characterize somatosensory involvement across studies, Michiels et al. (2018) conducted a global survey involving researchers, resulting in a consensus on a set of criteria for diagnosing somatosensory tinnitus. These included modulation of tinnitus by somatosensory system activation, and associations of tinnitus onset or aggravation with somatic comorbidities (such as temporomandibular joint and cervical spine disorders). As the purpose of the criteria are to standardize assessment of the extent to which the somatosensory system can influence an individual's tinnitus, no strict rules for the diagnosis of somatosensory tinnitus were proposed.

Moving to mental comorbidities, such as depression and anxiety, these have also been shown to commonly accompany tinnitus. Depression has been estimated between 14% (Stobik et al., 2005) and 80% (Hiller and Goebel, 2001) among tinnitus patients, correlating moderately with tinnitus-related distress (Langguth et al., 2011a). Additionally, several symptoms such as irritability, social withdrawal, worry, and insomnia overlap between the two conditions. Previous studies also highlighted the potential neural and genetic mechanisms that may account for tinnitus and depression (Langguth et al., 2011b). With regards to anxiety disorders, its lifetime prevalence among tinnitus patients has been estimated at 45% (Pattyn et al., 2016). This includes conditions such as generalized anxiety disorder, social and specific phobia, post-traumatic stress, and panic disorder. A dysfunctional link between the limbic and auditory system has been implicated as a potential neural mechanism for the co-occurrence of the two conditions, but the causal mechanisms linking anxiety and tinnitus remain unclear (Pattyn et al., 2016).

### 2.3. Challenges and Future Directions

This section discussed various dimensions of tinnitus heterogeneity, however, not in an exhaustive way. For example, tinnitus heterogeneity can also be explored in terms of differences in onset-related characteristics, modulating factors and other temporal and perceptual characteristics of tinnitus (e.g., tinnitus being maskable and/or prone to residual inhibition or not), and characteristics of response to specific treatments (Noreña et al., 1999; Koops et al., 2019; Simoes et al., 2019). Some of these sources of heterogeneity are touched upon in the following sections. Overall, any phenotypic trait of an individual with tinnitus can contribute to tinnitus heterogeneity. The biggest challenge is to identify the traits that could help differentiate subgroups of patients with different underlying mechanisms and treatment needs. Although many studies have addressed this question, reviews of existing literature would be very beneficial. Another widely accepted approach in tackling this task is to create a large database of high-quality tinnitus-specific data. Ideally, this would include detailed tinnitus-profiling information, such as brain imaging, genetic profiling, and treatment response history. Efforts should be made to collect information from diverse samples, covering the full range of tinnitus severity, to ensure all aspects of tinnitus heterogeneity are captured. Statistical analyses could then be conducted on this large dataset to address many of the unanswered research questions regarding tinnitus heterogeneity. Such methodologies have been successfully applied for other medical conditions such as diabetes and Alzheimer's disease (Ahlqvist et al., 2018; Mitelpunkt et al., 2020).

Collating such large data, however, requires a lot of time and funds. It is therefore becoming clear to the scientific community that interdisciplinary collaborations are essential. These should focus on standardization of tinnitus assessment, which would allow the creation of large tinnitus-specific databases, allowing the comparison between various datasets (Langguth et al., 2007; Hall et al., 2018c). The TRI database is a noteworthy example as it contains information from more than 5000 tinnitus patients from different centers (Landgrebe et al., 2010). It is important that such efforts should not only focus on clinically-relevant tinnitus cases (Landgrebe et al., 2010), but also population-based cohorts like the Swedish Tinnitus Outreach Project (STOP, 2015), allowing a comprehensive exploration of tinnitus heterogeneity. Other recent initiatives include the ESIT, the TIN-ACT, and the UNITI European Union funded projects, aiming to further integrate clinical data from different specialized centers in Europe, but also information from the general population. Such projects would allow a much needed multicenter comparison of the profile of tinnitus patients.

## 3. Epidemiology, Risk Factors, and Prevention

The prevalence of tinnitus shows widespread variability, with most studies reporting prevalence estimates between 10 and 15% (Baguley et al., 2013; Gallus et al., 2015; McCormack et al., 2016). In the UK alone, 0.5 to 1% of the population experiences clinically-significant tinnitus (Davis and El Refaie, 2000).

Available information on the epidemiology of tinnitus is centered around Western Europe, USA, and Australia (McCormack et al., 2016). Prevalence studies often include sample populations that are restricted to certain geographic areas or by specific demographics. For example, the Epidemiology of Hearing Loss Study (EHLS) is a population-based cohort providing valuable information on hearing in older adults, aged 43 to 84 years, conducted in Beaver Dam, Wisconsin, USA (Cruickshanks et al., 1998). Given the specific population and geographical characteristics, it might not be ideal to generalize results from such studies (Westreich et al., 2019).

Additionally, using a variety of assessment questions and/or research definitions across studies inconsistently gives rise to widespread variability in prevalence estimates (McCormack et al., 2016). For example, tinnitus can also include cases of sudden onset, lasting only a few seconds. However, these are not considered pathological (Levine and Oron, 2015). To exclude such cases, many studies define tinnitus as the presence of sound in the head or ears lasting for more than 5 min at a time (McCormack et al., 2016). However, a consensus on a standardized tinnitus definition is yet to be reached.

Tinnitus has been associated with many risk factors such as otological and psychological conditions, infectious diseases affecting the ear, hypertension, diabetes, systemic lupus erythematosus, rheumatoid arthritis, trauma, medications, noise exposure, and other lifestyle-related factors and environmental exposures (Han et al., 2009; Baguley et al., 2013; Biswas and Hall, 2020). Knowledge of tinnitus-related risk factors is necessary to identify vulnerable populations and implement adequate preventive measures to reduce the condition's burden. However, some risk factors lack a clear relationship with tinnitus. For example, although hearing loss has been reported to increase the risk for developing tinnitus in multiple studies (Nondahl et al., 2002; Baguley et al., 2013; Moore et al., 2017), the relationship between the two conditions is complex, as previously discussed. This inadequate understanding complicates the implementation of preventive measures.

Certain factors related to an individual's lifestyle (such as smoking and exposure to loud music) are known to increase the risk of developing tinnitus and avoiding them would decrease the risk (Zhao et al., 2010; Veile et al., 2018; You et al., 2020). Analytical observational studies (case-control and cohort) on tinnitus and potential lifestyle-related risk factors can help form inferences on causal associations. Unfortunately, in tinnitus research, most information is derived from cross-sectional data and there is a scarcity of longitudinal studies to assess the relationship between potential risk factors and tinnitus (Baguley et al., 2013).

Prolonged exposure to loud noises over time has been identified as a main risk factor for the development of tinnitus in individuals of all ages. Therefore, the need for educational preventive programs focusing on ear protection are paramount (Williams et al., 2010). The challenge of measuring the efficacy of this type of intervention lies in the associated costs and the time needed for the programs to have an impact, thus receiving low priority. Consequently, there is a lack of empirical studies evaluating the feasibility and implementation (e.g., classroom vs. internet-based) of such preventive programs.

Several steps can be undertaken to tackle the challenges discussed in tinnitus epidemiological research. Firstly, addressing the lack of a standard definition and developing unambiguously phrased research questions are crucial for understanding tinnitus epidemiology. To ensure uniformity in assessment and for ease of comparison between populations, there is a clear need for cross-culturally adapted assessment questions using good practice guidelines (Hall et al., 2018e), instead of verbatim translations. To address this issue, Biswas et al. (2019) proposed a set of standardized questions on tinnitus prevalence and severity in 12 European languages following good practice guidelines for translating hearing-related questionnaires (Hall et al., 2018e). Secondly, there is an evident geographical bias with tinnitus prevalence studies predominantly emanating from a limited number of regions (McCormack et al., 2016). To have a better global perspective, it is important to encourage epidemiological research in other regions. Thirdly, conducting analytical observational studies as well as exploring existing data sets might reveal new and significant associations between tinnitus and potential risk factors. Lastly, there remains a clear need to evaluate the feasibility and efficacy of preventive measures. Addressing knowledge gaps in future studies and synthesizing existing knowledge, using systematic literature reviews, could lead to significant progress in tinnitus epidemiological research.

## 4. Tinnitus Mechanisms

### 4.1. Overview

Theories and experimental evidence on tinnitus pathophysiology are rather diverse as highlighted by a systematic review (Haider et al., 2018). The site of tinnitus generation is still debated, with the potential involvement of both peripheral and central structures. For example, Noreña (2015) proposed differentiating three subtypes of tinnitus: cochlear (abnormal activity at the peripheral auditory system), peripheral-dependent central (tinnitus neural activity in higher centers but dependent on peripheral activity), and peripheral-independent central tinnitus (tinnitus neural activity in higher centers independent of peripheral activity). According to another model based on predictive coding, the presence of a tinnitus precursor in the form of spontaneous activity at subcortical auditory pathways was speculated (Sedley et al., 2016). It was suggested that this spontaneous activity is normally ignored by higher cortical structures, but increase in its precision gives rise to tinnitus. In the following sections, evidence and models for the involvement of hearing-related and other neural structures, as well as genetics in tinnitus, are discussed. Additionally, key challenges and future directions for these research domains are highlighted.

### 4.2. Hearing Deprivation and Brain Plasticity

The brain has the capability to transform its existing circuitry and functions in response to intrinsic or extrinsic stimuli, which is referred to as brain plasticity or neural plasticity (Mateos-Aparicio and Rodríguez-Moreno, 2019). This ability is subject to homeostatic regulation to maintain an environment where it can function optimally (Turrigiano and Nelson, 2004; Turrigiano, 2008). However, deprivation of auditory input can cause a harmful plasticity effect that could lead to the onset of tinnitus (Noreña and Farley, 2013). This is supported by the finding that tinnitus may disappear when hearing recovers (Schreiber et al., 2010).

Disruptions from lower levels of the auditory pathway could lead to alterations in synaptic transmission and neurotransmitter release in more central areas of the auditory system. This creates an imbalance between neuronal excitation and inhibition and re-routing of auditory pathways, leading to overall abnormal neural activity (Møller, 2007; Shore et al., 2016). More specifically, reduction of the main inhibitory neurotransmitter, GABA (gamma-aminobutyric acid), has been associated with tinnitus (Yang et al., 2011; Sedley et al., 2015). Changes in GABA neurotransmission may dysregulate the necessary inhibition controlling spontaneous neural activity in the auditory system. The abnormal neural activity linked to the tinnitus percept involves not only the auditory system as thought previously, but also non-auditory neural networks including networks of the sensory, cognitive, and affective systems (Rossiter et al., 2006; Adjamian et al., 2009; De Ridder et al., 2011; Schmidt et al., 2013; Leaver et al., 2016). However, the current evidence is too spread out, indicating neural activity abnormalities in different parts of the brain. For an in-depth review, see Lanting et al. (2009) and Husain and Schmidt (2014).

### 4.3. Neuroimaging

Neuroimaging attempts to identify putative models that underlie tinnitus emergence and the persistent nature of the perception. In recent years, several neuroimaging techniques have succeeded in finding structural, functional and neurochemical changes in individuals with tinnitus (Adjamian et al., 2014; Elgoyhen et al., 2015; Sedley et al., 2015; Shahsavarani et al., 2019; Adams et al., 2020).

Various brain imaging studies using voxel-based morphometry (VBM), diffusion tensor imaging (DTI), functional magnetic resonance imaging (fMRI), and positron emission tomography (PET) revealed structural and functional changes associated with tinnitus across distributed auditory and non-auditory neural networks. Identified regions of interest include areas of the auditory system (e.g., the primary and secondary auditory cortices, medial geniculate body, and inferior colliculus), the limbic system (e.g., the anterior cingulate cortex, amygdala, insula, parahippocampus, and nucleus accumbens), attention-related networks (e.g., the occipito-parietal cortex and supramarginal cortex), and the default mode network (e.g., medial prefrontal cortex and precuneus) (Adjamian et al., 2014; Elgoyhen et al., 2015; Raichle, 2015; Sedley et al., 2015; Shahsavarani et al., 2019; Adams et al., 2020). In particular, reduced gray matter (Landgrebe et al., 2009; Schneider et al., 2009; Aldhafeeri et al., 2012) and changes in activity (Arnold et al., 1996; Langguth et al., 2006; Boyen et al., 2014) in auditory system areas have been linked to tinnitus perception. Abnormal structural (Crippa et al., 2010; Besteher et al., 2019) and functional (Schmidt et al., 2013; Davies et al., 2017) changes of the amygdala and parahippocampus have also been associated with tinnitus. Moreover, imaging studies have revealed structural and functional abnormalities in the anterior cingulate cortex and the insula (Carpenter-Thompson et al., 2014; Chen et al., 2018; Besteher et al., 2019). Additionally, decreased brain matter volume (Aldhafeeri et al., 2012) in the default mode network and changes in functional connectivity (Schmidt et al., 2013; Chen et al., 2018) in the default mode and attention networks may play a pivotal role in neuropathological features underlying tinnitus. However, results from structural and functional neuroimaging are highly heterogeneous, which could be due to small sample sizes, lack of standardized sample selection criteria and heterogeneity of sample characteristics (e.g., degree of hearing loss and presence of psychiatric comorbidities), and differences in imaging and data analysis methods. More in-depth reviews on tinnitus structural and functional neuroimaging can be found in Adjamian et al. (2014), Elgoyhen et al. (2015), and Adams et al. (2020).

Further, neurophysiological investigations using electroencephalography (EEG) or magnetoencephalography (MEG) were able to identify tinnitus-associated pathological spontaneous brain activity patterns. In particular, enhanced activity in the gamma and delta frequency range along with reduced alpha activity over the auditory cortices was observed in tinnitus patients (Weisz et al., 2005, 2007b; Moazami-Goudarzi et al., 2010; Adjamian et al., 2012; Balkenhol et al., 2013; Schlee et al., 2014). These oscillatory aberrations were subsumed under the framework of the thalamo-cortical dysrhythmia model (TCD) (Llinás et al., 1999, 2005; De Ridder et al., 2015) and further extended to the synchronization by loss of Inhibition model (SLIM) (Weisz et al., 2007a). However, results of other studies are not in accordance with these observations (Ashton et al., 2007; Moazami-Goudarzi et al., 2010; Meyer et al., 2014; Pierzycki et al., 2016), emphasizing instead on the importance of other frequency bands in neural activity related to tinnitus (Moazami-Goudarzi et al., 2010; Balkenhol et al., 2013; Meyer et al., 2014). In view of these ambivalent findings, it still remains unclear how and which altered ongoing brain activity patterns are associated with tinnitus.

To observe the brain's neurochemical characteristics in tinnitus, magnetic resonance spectroscopy (MRS) can be used. MRS data is taken using an MRI scanner, producing a graphical spectrum with peaks corresponding to different chemical concentrations from voxels of interest (McRobbie et al., 2017). As discussed earlier, altered GABA levels were observed in tinnitus patients with hearing loss (Sedley et al., 2015). Confirmatory evidence of decreased GABA inhibition was also seen in patients with hearing loss alone (Gao et al., 2015), which might suggest that reduced GABAergic tone may directly result from the loss of peripheral input. Following these results, additional research is required in order to observe the exact role of different neurotransmitters and how big are the changes related to tinnitus specifically. Other neurotransmitters such as glycine and glutamate are also well-associated with signaling mechanisms of the central auditory pathway (Lee and Godfrey, 2015) and thus also need to be investigated in future research studies.

To date, both functional and structural findings have been very inconsistent with little replication, potentially due to the heterogeneity of tinnitus characteristics, relatively small sample sizes, and variability in data acquisition and analysis methods. Conducting and updating systematic reviews of methods and results of previous studies is essential in order to understand common patterns and inconsistent findings, guiding future studies. An important consideration for future work is to dissociate findings that are tinnitus-specific, rather than due to hearing loss or hyperacusis. In order to reliably identify the main neural structures and pathways related to the various aspects of tinnitus, carefully designed studies with detailed phenotyping of recruited participants (both cases and controls), and large sample sizes for sufficient statistical power (Button et al., 2013) need to be conducted. Standardization in methodological aspects of the aforementioned techniques would additionally facilitate comparing findings across studies.

### 4.4. Tinnitus Genetics

Research related to the genetic components of tinnitus is sparse (Vona et al., 2017). Nevertheless, it has been speculated that tinnitus is related to a multifactorial genetic etiology (Lopez-Escamez et al., 2016). To study the genetic contribution to diseases, various study designs can be implemented including heritability twin studies and linkage analysis (Sahebi et al., 2013). To investigate the genetic susceptibility to tinnitus, a study was conducted on a Swedish twins cohort of 10,464 pairs with self-reported tinnitus (Maas et al., 2017). A higher rate of concordance was reported in monozygotic pairs compared to dizygotic. Heritability was 56% for bilateral tinnitus compared to 27% for unilateral tinnitus and further stratification by sex showed an increased heritability for males (68%). Additionally, a study on Swedish adoptees with clinically significant tinnitus showed that the shared environment was not associated with tinnitus presence (Cederroth et al., 2019b).

In order to explore the association between common genetic variants across the whole genome and a phenotype of interest, genome-wide association studies (GWAS) are considered a robust approach (McCarthy et al., 2008). These are hypothesis-free, case-control linkage studies, that have been successful in revealing genetic mechanisms of common diseases such as diabetes (Todd et al., 2007), inflammatory bowel diseases (Parkes et al., 2007), major depression (Wray et al., 2018), and breast cancer (Easton et al., 2007). A pilot GWAS on tinnitus was performed in an adult population (55-65 years old) of a homogeneous ethnic background from Belgium, comprising of 167 self-reported tinnitus cases and 794 controls (Gilles et al., 2017). None of the single nucleotide polymorphisms (SNPs) reached genome-wide significance, possibly due to the small sample size. However, a subsequent gene-set enrichment analysis asking whether any biological pathway is significantly associated with the results of the GWAS revealed seven metabolic pathways such as involvement of endoplasmic reticulum stress, NRF2-mediated oxidative stress response, and serotonin receptor mediated signaling pathways (Gilles et al., 2017). GWAS meta-analysis is a solution to overcome the restrictions of the sample size and investigate potential sources of heterogeneity. Additionally, a candidate-gene approach can be used to look at the variation associated with a disease within a number of predefined genes. Such candidate-gene studies have investigated the role of genes such as BDNF (Sand et al., 2012), GDNF (Orenay-Boyacioglu et al., 2016), KCNE1, and SLC12A2 (Pawełczyk et al., 2012) in tinnitus development, susceptibility, and severity.

Several challenges emerge during attempts to map the genetic architecture of tinnitus. First, due to the lack of a clear Mendelian pattern of inheritance, tinnitus is considered to be a complex trait. Such complex traits are difficult to link to a specific genetic marker due to several reasons such as incomplete penetrance, genetic heterogeneity, and polygenic inheritance (Lander and Schork, 1994; Brown et al., 2019). Second, the self-reported nature of tinnitus diagnosis (due to the lack of a biomarker) and the lack of standardization for defining tinnitus and tinnitus subgroups can lead to an inclusion of heterogeneous tinnitus cases, affecting results of any subsequent analysis. Lastly, tinnitus can be associated with many other disorders including Meniere's disease, hearing loss, depression and sleep disorders, making it difficult to clearly define the independent genetic component of tinnitus.

To overcome these challenges, like in the case of neuroimaging studies, careful selection of tinnitus cases with adequate phenotyping and sufficient sample sizes in future studies are essential (Riley et al., 2020). In addition, specific study designs can help overcome some of these challenges. For example, genetic association studies with a case-control design and a candidate-gene approach looking at the variation associated with tinnitus within a number of predefined genes can be conducted (Lopez-Escamez et al., 2016). Another methodology that can prove useful is extreme phenotype design, which is a case-control study design with the assumption that the furthest cases on both extremes of a phenotype distribution can play an important role in identifying rare variants and candidate genes contributing to a particular trait. Using this strategy, sequencing a moderate sample size is enough to find enrichment of rare variants in extremes of a quantitative trait (Bamshad et al., 2011).

## 5. Tinnitus Assessment

### 5.1. Overview

Despite efforts for standardization, as of yet there is no universally accepted protocol for tinnitus assessment for either clinical or research settings (Langguth et al., 2006; Henry, 2016). Nevertheless, clinicians and researchers tend to agree that many pieces of information are essential in order to sufficiently characterize an individual with tinnitus (Langguth et al., 2006; Levine and Oron, 2015). Table 1 presents an overview of elements for tinnitus assessment summarized from selected sources (Langguth et al., 2007; Tunkel et al., 2014; Levine and Oron, 2015; Durai and Searchfield, 2016; Fuller et al., 2017; Cima et al., 2019) and/or used from the authors of this article. Across tinnitus literature, even more variables have been assessed for their relevance for tinnitus profiling such as biochemical markers (Ban et al., 2018). Neuroimaging and genetic profiling have been discussed in previous sections. The focus of this section is tinnitus profiling based on case history and audiological assessment. Which of these elements should be included in any tinnitus assessment protocol for clinical and/or research settings remains to be established.

**Table 1:** Overview of elements for tinnitus assessment.

**Elements**	**Examples of specific measures**
**Non tinnitus-specific**	
General individual characteristics	
Sociodemographic, lifestyle characteristics	Standardized tinnitus case history questionnaires such as the TSCHQ and the ESIT-SQ
Personality traits	Personality questionnaires such as the Eysenck personality inventory (Eysenck and Eysenck, 1964; see Durai and Searchfield, 2016 for a review)
Noise and other exposures	Structured interview or self-reported questionnaires for noise exposure (see Guest et al., 2018 for a review)
Ear and hearing-related conditions	
Ear, noise, and throat conditions	Ear, noise, and throat assessment
	(complete ear, nose and throat clinical examination including otoscopy, auscultation of head and neck area for audible sounds, palpation of head and neck area for masses or trigger points, examination of the temporomandibular joint)
Auditory and vestibular function	Audiological assessment
	(standard air conduction and bone conduction PTA, speech audiometry, immittance tympanometry, acoustic reflex assessment, auditory brainstem responses, high frequency PTA, otoacoustic emission, loudness discomfort levels, caloric testing, vestibular evoked myogenic potential), self-report questionnaires for assessing hearing disabilities such as the SSQ
Vertigo, hyperacusis, and other hearing and vestibular comorbidities Clinical examination, structured interview, or self-report case history questionnaires such as the ESIT-SQ, self-report questionnaires for assessing hyperacusis (Hyperacusis Questionnaire from Khalfa et al., 2002; see Margol-Gromada et al., 2020 for an overview), psychoacoustic ratings of natural sounds (Enzler et al., 2021)	
Psychological conditions (e.g., mood disorders, anxiety disorders, reaction to severe stress)	Psychological and/or psychiatric assessment, medical records review, structured interview, self-report questionnaires for psychological disorders such as the Hospital Anxiety and Depression Scale and the Beck Depression Inventory (Zigmond and Snaith, 1983; Beck et al., 1988; see Durai and Searchfield, 2016 for a review)
Treatment history (e.g., medication and procedures)	Medical records review, structured interview, or self-report questionnaires such as the ESIT-SQ
Other medical history and relevant co-existing conditions (e.g., dental conditions, cervical conditions, headaches, neurological conditions, cognitive abilities, pain syndromes, overall quality of life)	Physical examination by relevant clinicians (e.g., dentist, physiotherapist, neurologist), cognitive-attention tasks, medical records review, structured interview, or self-report questionnaires such as the ESIT-SQ for general comorbidities or the WHOQOL-BREF (WHO, 1998) and EQ5D (Herdman et al., 2011; Stolk et al., 2019) for quality of life assessment.
Brain anatomy and function	Electroencephalography (EEG), magnetoencephalography (MEG), magnetic Resonance Imaging (MRI), positron emission tomography (PET)
Genetic profile	Isolated DNA from saliva.
**Tinnitus specific**	
Tinnitus history Onset related characteristics including duration, age at onset, onset related events, and sudden or gradual onset Presence pattern including frequency of presence, and being constant or intermittent Other perceptual characteristics including quality, being pulsatile, pitch, spatial perception, variability (e.g., loudness fluctuation) Modulating factors (e.g., somatic modulation)	Structured interview or self-report questionnaires such as ESIT-SQ
Tinnitus Impact	Structured interview (Tunkel et al., 2014; Cima et al., 2019) or self-report questionnaires such as the Tinnitus Handicap Inventory (Newman et al., 1996), the Tinnitus Functional Index (Meikle et al., 2012), the Tinnitus Handicap Questionnaire (Kuk et al., 1990), and the Tinnitus Reaction Questionnaire (Wilson et al., 1991) (see Tunkel et al., 2014; Durai and Searchfield, 2016; Cima et al., 2019 for reviews)
Tinnitus psychoacoustic assessment Pitch matching Loudness matching Minimum Masking Level (MML) Residual inhibition	As in Fournier et al., 2018; Neff et al., 2017; Hoare et al., 2014a; Roberts, 2007

*ESIT-SQ, European School for Interdisciplinary Tinnitus Research Screening Questionnaire (Genitsaridi et al., 2019); PTA, Pure Tone Audiometry; SSQ, Speech Spatial and Qualities of Hearing Scale (Gatehouse and Noble, 2004); TSCHQ, Tinnitus Sample Case History Questionnaire (Langguth et al., 2007)*.

### 5.2. Case History Assessment

Numerous characteristics of patient case history have demonstrated importance for tinnitus profiling, including demographics, co-existing conditions, and tinnitus perceptual characteristics (van de Heyning et al., 2014). Ideally, such information should be collected using standardized interviews, clinical examinations, diagnostic tests and medical record assessment to ensure data quality. However, for practical reasons, they are often self-reported and as they can be assessed with standardized questionnaires (Langguth et al., 2017; Genitsaridi et al., 2019). For conditions that cannot be sufficiently assessed with single questions, such as personality traits, psychological conditions, hyperacusis and noise exposure history, specific multi-item questionnaires have been developed (Khalfa et al., 2002; Durai and Searchfield, 2016; Guest et al., 2018).

An essential part of tinnitus assessment is the measurement of tinnitus severity and more generally, the impact of tinnitus on the affected individual which can vary largely from one individual to another (Hall et al., 2018a). Such measurement is also essential for assessing the effectiveness of a tinnitus intervention. Numerous multi-item self-report questionnaires have been developed for this purpose (Haider et al., 2016), such as the Tinnitus Handicap Inventory (THI) (Newman et al., 1996) and the Tinnitus Functional Index (TFI) (Meikle et al., 2012). Visual analog scales and numeric rating scales are also commonly used instruments to quantify tinnitus loudness and distress (Adamchic et al., 2012).

Even though previous recommendations have been made regarding which instruments to use for assessing the impact of tinnitus and treatment efficacy (Langguth et al., 2007; Jacquemin et al., 2018), there is no consensus yet in the tinnitus scientific community and new instruments are still being developed (Tyler et al., 2014a).

### 5.3. Audiological Assessment

Tinnitus profiling cannot be complete without an assessment of the auditory system. Although sometimes self-reported instruments are used to collect information about subjective hearing problems, hyperacusis, noise exposure history, and other hearing-related conditions, an audiological assessment remains essential. This usually includes hearing and tinnitus psychoacoustic assessment.

#### 5.3.1. Hearing Assessment

As discussed previously, hearing assessment often involves only standard PTA. However, a more comprehensive hearing assessment, including higher frequency resolution and extended high frequencies, would benefit tinnitus profiling as it would provide higher sensitivity for auditory system impairments (Vielsmeier et al., 2015; Lefeuvre et al., 2019; Xiong et al., 2019). Besides PTA, numerous other audiological tests can be used for a more in depth characterization of hearing function, including speech in noise audiometry, immittance tympanometry, acoustic reflex assessment, auditory brainstem responses (ABR), otoacoustic emissions (mainly distortion product otoacoustic emissions [DPOAE]), and loudness discomfort levels. However, the more tests added to an assessment protocol, the more time required to collect the desired information per participant. Therefore, researchers need to consider if more detailed hearing assessments would be beneficial for their specific studies, thus being critical when deciding which measures to include in their assessment protocols. One solution to reduce testing time while gaining additional information is the use of machine learning techniques, for example, as applied in audiometry (Schlittenlacher et al., 2018). In this case, the sound trials tested would be those with higher uncertainty, lowering the total number of trials tested and, consequently, the duration of testing. Furthermore, the procedure can be automatized relieving the audiologist from the task.

#### 5.3.2. Tinnitus-Specific Psychoacoustics

The subjective nature of tinnitus requires further tinnitus-specific psychoacoustic assessment. An adequate matching of pitch and loudness can help better understand the underlying mechanisms of tinnitus perception and to ensure correct application of sound-based therapies on an individual level (Schaette et al., 2010; McNeill et al., 2012; Pantev et al., 2012). For pitch matching various methods have been investigated. For example, methods like the “2AFC” (Penner and Bilger, 1992), the “likeness rating” (Noreña et al., 2002) or the “method of adjustment” (Wier et al., 1976) have been broadly used in previous studies. These methods vary in their reliability, duration or subjective satisfaction of participants, and future research should try to combine their strengths (Neff et al., 2019a). With regards to loudness, the most common methods of assessment are loudness matching and loudness rating. Loudness matching can be done at the tinnitus frequency or at prespecified frequency such as at 1 kHz, by presenting and adjusting the intensity of an external sound until it is perceived as equal to tinnitus loudness (Mitchell et al., 1993; Hoare et al., 2014a; Hall et al., 2017). The matched loudness can be expressed in either dB HL (hearing level) or in dB SL (sensation level, i.e., the difference between the identified loudness level and the hearing threshold at the given frequency). Learning effects seem to play a main role in the subjectivity of the assessment (Hoare et al., 2014a). In addition, loudness matching has been shown to not correlate with loudness ratings, which better describes tinnitus intrusiveness and severity and provide more useful information from a clinical perspective (Henry, 2016).

Regarding tinnitus maskability, it is known that some tinnitus patients report that external sounds can not mask their tinnitus, while others report that even weak sounds at any frequency mask it (Henry and Meikle, 2000). A common method to assess tinnitus maskability is the minimum masking level (MML), which is the lowest level of a noise required to mask tinnitus, and it is a useful tool to measure the intrusiveness of tinnitus and the acceptance of masking (Vernon et al., 1990; Andersson, 2003). Other methods of masking aim to target the tinnitus frequency, such as the Tinnitus Tuning Curve (TTC). The TTC assesses the maskability of tinnitus at different frequencies and levels. Fournier et al. (2019) investigated differences in perception of external sounds and tinnitus, and compared TTC and Psychophysical Tuning Curves (PTC) in people with tinnitus. Their results indicated the presence of different subgroups within the whole cohort.

Another psychoacoustic measure routinely assessed in the clinic is residual inhibition (RI). It can be defined as the temporary suppression of tinnitus that occurs following exposure to a noise presented for 30 s or 1 min at a level 10 dB above the tinnitus masking threshold (the minimum level required to mask tinnitus or minimum masking level) (Feldmann, 1971, 1983; Vernon and Meikle, 2003; Roberts, 2007). This phenomenon is observed in approximately 60–80% of tinnitus patients. Suppression patterns appear to vary across individuals and are dependent on stimulation intensity, duration and/or frequency (Roberts et al., 2006, 2008; Schaette et al., 2010; Neff et al., 2017). A new method has recently been developed by Fournier et al. (2018), where the measure of RI duration is replaced by a measure of minimum sound intensity, which allows RI for a short and fixed time interval (1 s). The stimulation used for the measures consisted of pulsed narrowband noises of a 3-s duration followed by 1-s silence intervals between the pulses. MML was obtained by increasing the intensity of the narrowband noise until the tinnitus was masked during the 3-s noise presentation, and the minimum residual inhibition level (MRIL) by further increasing the stimulus intensity until the tinnitus was suppressed during the silence period between the acoustic pulses. Besides being less time-consuming than the classic method, this method was proven to provide minimum masking and residual inhibition levels in 98.5 and 86.7% of the patients, respectively. However, the reliability and reproducibility of the measurements obtained with this method remain to be verified. Furthermore, by investigating the consequences of various modulated or filtered sounds (Reavis et al., 2012; Henry et al., 2013; Tyler et al., 2014b; Neff et al., 2017, 2019b; Schoisswohl et al., 2019), the groundwork for new potential long term sound therapies can be laid.

### 5.4. Challenges and Future Directions

One of the main challenges in tinnitus assessment is to define a minimum set of essential information to collect. For clinical practice, the aim would be to have enough information pointing to a specific etiology that would require specific treatment (Cima et al., 2019). For example, a person with tinnitus, no comorbidities, audiometrically normal hearing and neck problems would require a different approach than a person with tinnitus and moderate hearing loss without other comorbidities or a person with tinnitus and depression. Similarly, in a research setting, the aim would be to define homogeneous populations with common underlying pathophysiology. In both cases, this minimum set of information can only be finalized once questions about tinnitus mechanisms and heterogeneity are answered.

Another important challenge in tinnitus assessment is the standardization of measures that would enable comparisons between independent research findings. For all the previously mentioned aspects of tinnitus assessment (including self-reported information and audiological assessment), a plethora of measures for data collection exist. This is also true for measures of treatment outcome, as is further discussed in section 6.6. Similar to the previously discussed tinnitus domains, systematic reviews to identify the most relevant and reliable measures for tinnitus assessment are essential. In addition, it is clear that for tinnitus research to move forward, a consensus for tinnitus assessment involving researchers from various fields is required, and such efforts are ongoing (Hall et al., 2018c; Schlee et al., 2018).

Given the challenges in tinnitus assessment, the need for identifying an objective measure for tinnitus is evident. Measures such as genetic, neuroimaging, biochemical and audiological markers, are prime candidates for objectively measuring the presence of tinnitus or of a particular tinnitus subtype. A recent systematic review could not identify any reliable objective measure for tinnitus but discussed some emerging areas of research; blood and neuroimaging tests, for instance (Jackson et al., 2019). Other studies discussed the role of Middle Ear Muscle Reflex (MEMR), also known as Acoustic Stapedial Reflex (ASR), as an objective tinnitus marker, however, with contradicting results (Wojtczak et al., 2017; Guest et al., 2019). Further research is required toward establishing the value of existing markers for objectively measuring tinnitus or toward identifying new markers.

## 6. Treatment Development

### 6.1. Overview

Although tinnitus is a commonly reported condition, the available treatment options are limited and aimed at managing tinnitus, rather than eliminating the percept (McFerran et al., 2019). From a clinical standpoint, the attenuation of the tinnitus percept and/or burden is a successful goal. In this context, the tinnitus-related burden can also be attenuated by managing comorbidities (Tunkel et al., 2014). Since tinnitus is a heterogeneous condition, and the perception of one's tinnitus is entirely subjective, the current efficacy of treatments varies. There are currently two main categories of treatment options; sound-based and psychology-based treatments (Tunkel et al., 2014; Fuller et al., 2017). Although there are currently no medications approved for tinnitus, pharmacology-based interventions are also under investigation. Research, challenges and future directions regarding these interventions and other therapeutic approaches, such as non-invasive neurostimulation, neurofeedback, and tinnitus mobile phone applications, will be discussed in the following sections.

### 6.2. Psychology-Based Interventions

Psychology-based approaches, including counseling and cognitive behavioral therapy (CBT), can help manage how tinnitus impacts emotions and behavior (Thompson et al., 2017; Landry et al., 2020). CBT comprises various components (e.g., relaxation, exposure, psychoeducation) which aim at changing cognitive, behavioral and emotional responses to tinnitus. It has been used for tinnitus treatment with positive results such as decreased tinnitus disability (Cima et al., 2012). A recent Cochrane review on CBT for tinnitus (Fuller et al., 2020) corroborates the reduction of the negative impact of tinnitus on quality of life. Moreover, CBT for tinnitus treatment received the highest level of recommendation by a multidisciplinary group who reviewed thirteen current treatment options (Cima et al., 2019). Despite the benefits of CBT, treatment is not always delivered to those who need it (Cima et al., 2020). In order to bridge the gap, internet-based CBT (iCBT) is currently being developed to facilitate the delivery to patients (e.g., Beukes et al., 2018). While a full iCBT treatment proves difficult, current research also focuses on dismantling treatment components to understand which parts work best for whom (e.g., Lourenco 2019). As such, future research aims at tailoring CBT to reduce the time of treatment and increase positive outcomes as well as exploring alternative platforms for delivery, such as online applications.

### 6.3. Sound-Based Interventions

#### 6.3.1. Sound Generators

Sound-based interventions include use of electronic devices such as sound generators, hearing aids, and cochlear implants. The principle of sound generators, which were developed decades ago, was to distract tinnitus patients with a more pleasant and acceptable sound, rendering tinnitus inaudible (Vernon, 1976). It is still not fully understood whether the effect of sound generators is by masking (Henry et al., 2008), interactions with putative tinnitus mechanisms (Jastreboff and Hazell, 1993), or affecting cognitive mechanisms such as attention or stress management. Tailor-made notched music training (TMNMT) is a sound-therapy approach based on the long-term application of a signal where the frequencies around the tinnitus pitch are filtered out. This is thought to reverse maladaptive reorganization of auditory cortical areas (Okamoto et al., 2010; Pantev et al., 2012). Overall, many studies have shown a positive effect of sound generators with tinnitus subjects experiencing some relief in tinnitus annoyance (Sereda et al., 2018). However, very few included a placebo condition, and the vast majority have included other components in the treatment, such as amplification or counseling (Hobson et al., 2012; Sereda et al., 2018). This has made it very difficult to evaluate the effect of the sound generators. It is therefore essential that future studies solely focus on assessing the effectiveness of various sound generators in well-designed randomized controlled trials (RCTs). Furthermore, the wide range of outcome measures used in these studies make comparisons difficult, and it is recommended that future studies use more meticulous methodology along with proper blinding and randomization, to secure high-quality evidence (Sereda et al., 2018).

#### 6.3.2. Hearing Aids

Hearing aids can also be used for tinnitus management, but the mechanisms of their effect are complex and not fully understood, as in the case of sound generators. They might have physiological effects on brain activity by reversing or preventing the maladaptive plasticity (Noreña, 2011), or by decreasing the neuronal response gain (Schaette et al., 2010). Moreover, people with hearing loss and tinnitus might also benefit from hearing aids due to the masking and distraction from tinnitus that they can provide (Sereda et al., 2015), and by improving their communication which could lead to a reduction in stress and anxiety. Although hearing aids have been used in tinnitus management since the 1940s (Saltzman and Ersner, 1947), evidence supporting their effectiveness is limited. Previous work has demonstrated the lack of evidence to support or refute hearing aids prescription in tinnitus management in patients with co-existing hearing loss (Hoare et al., 2014b). Shekhawat et al. (2013) highlighted the lack of high-quality evidence for the effect of hearing aids since most studies do not use RCTs. Moreover, limitations and differences in clinical guidelines for diagnosis and management of tinnitus make the assessment of this particular treatment more difficult (Sereda et al., 2015). Future research on hearing aids and tinnitus management must be carried out by well-designed RCTs under a standard framework for diagnosis and prescription. Besides general fitting procedures, assessing individual tinnitus characteristics could help personalize hearing aid fitting which could lead to increased effectiveness. For example, previous studies have suggested that there can be a higher reduction in tinnitus loudness and scores of the Tinnitus Reaction Questionnaire (TRQ) when the tinnitus pitch is located within the frequency range of the amplification (Schaette et al., 2010; McNeill et al., 2012). Approaches like TMNMT should be investigated in-depth for hearing aids. Furthermore, more focus has recently been put on individualized compensation strategies that consider additional parameters when fitting hearing aids to improve speech intelligibility (Vlaming et al., 2011; Santurette and Dau, 2012; Sanchez-Lopez et al., 2020). In the future, it would be interesting to develop unique auditory profiles and compensation strategies for tinnitus patients and investigate whether these more specialized fittings will improve the benefits of hearing aids on tinnitus. Combination aids can be used for both amplification and sound stimulation, and they might be an interesting option for some tinnitus patients. However, the available evidence is not sufficient, and recommendations on the type of noise, level of noise or laterality of fitting should be standardized to shed some light on their efficacy (Tutaj et al., 2018).

#### 6.3.3. Cochlear Implants

Over the years, many research groups have investigated the efficiency of cochlear implants (CI) that specifically aim to reduce the perception of tinnitus (Van de Heyning et al., 2008; Ramakers et al., 2015; Macías et al., 2018; Peter et al., 2019; Assouly et al., 2020). Although the methods used to objectively measure and record the tinnitus levels before and after the surgery, the number of patients in a study cohort and the follow up periods vary significantly among the research groups, these studies collectively show a clear improvement for tinnitus patients. However, like hearing aids, little is known about the involved mechanisms. One popular claim is the neural plasticity induced by intracochlear electrical stimulation provided by CIs (Fallon et al., 2008). However, after long term follow-up, CI recipients report that once the implant is turned off, the tinnitus comes back (Mertens et al., 2016). Additionally, in some cases worsening or induction of tinnitus has been reported (Quaranta et al., 2004; Baguley and Atlas, 2007; Di Nardo et al., 2007; Pan et al., 2009; Kompis et al., 2012). Due to this heterogeneity in outcomes, researchers have tried to develop models to predict positive and negative effects of cochlear implantation for tinnitus (Ramakers et al., 2018; Kloostra et al., 2019; Dixon et al., 2020), but further research needs to be carried out using prospective studies and extensive databases. Optimized CI programming for tinnitus patients is another important but under-researched domain (Pierzycki et al., 2019). Long term follow-up studies, standardization of outcome measures, and additional research are required to gather more evidence to convince medical authorities and decision-making bodies that CIs could be a successful treatment option for patients who suffer from severe and disabling tinnitus (Newman et al., 1996; Meikle et al., 2012; Hall et al., 2018b; McFerran et al., 2019). This approach must involve not only audiologists and ENT physicians, but also insurance bodies and policymakers to create a clear pathway to provide CI to patients seeking help for tinnitus.

### 6.4. Pharmacology-Based Interventions

A wide variety of therapeutic drugs have been used to relieve tinnitus (Elgoyhen and Langguth, 2010). For acute tinnitus, a dose-dependent reduction in tinnitus intensity was observed with intravenous lidocaine (Trellakis et al., 2006). However, its use is controversial due to its short-lasting response, its potentially life threatening arrhythmogenic side effects, and the low bioavailability of its oral form (Israel et al., 1982; Trellakis et al., 2007; Gil-Gouveia and Goadsby, 2009). A potential goal of pharmacologic tinnitus research could be to identify the mechanism by which lidocaine interferes with tinnitus and mimic this effect using a drug with better tolerance that can be orally administered. For chronic tinnitus, the off-label use of medicines like betahistine (Hall et al., 2018d), anticonvulsants (Hoekstra et al., 2011), and glutamate receptor antagonists have shown little or no effect in clinical trials. Prescription of antidepressants and benzodiazepines is limited to tinnitus-associated comorbidities such as depression, insomnia and anxiety (Langguth et al., 2019). Moreover, three clinical research programs, in the last few years, were discontinued in phase II and III. AMPA antagonist selurampanel (BGG492) has not resulted in a new compound (Cederroth et al., 2018). NMDA receptor antagonists (AM-101) did not meet the primary endpoint of improving minimum masking level in acute tinnitus in a phase II clinical trial but showed improvement for tinnitus loudness, annoyance, sleep difficulties, and tinnitus impact in patients with tinnitus after noise trauma or otitis media (van de Heyning et al., 2014). Many other treatments decreasing tinnitus percept or targeting central auditory processing pathways are at a preclinical phase (Schilder et al., 2019). The modulator of voltage-gated potassium channels (Kv3.1) (AUT00063) was not effective in alleviating tinnitus symptoms (Hall et al., 2019b).

The search for pharmacological treatments involves not only identification of targets (genes/proteins), but also routes of administration allowing optimal bioavailability. No clear endophenotype or neural substrate has been identified for tinnitus (Elgoyhen and Langguth, 2010). Tinnitus is described as a side effect of a non-negligible number of compounds (Campbell and Le Prell, 2018). The side-effect network integration revealed many potential targets involved in its generation (which confirms its heterogeneity) (Elgoyhen et al., 2014). Efforts for the development of otoprotective agents and gene therapies for gene correction and hair cells regeneration in sensorineural hearing loss (SNHL) have been increasing with four programs in preclinical phase and one in clinical development (Phase I/II) (Schilder et al., 2019). Besides, intratympanic injections can be used for efficient and reliable drug delivery. They remediate for the loss of effectiveness of systemic routes of administration (oral or intravenous) due to bioavailability due to the blood-labyrinth barrier and have reduced side effects. Formulation strategies including hydrogels, polymers and nanoparticulate systems could potentiate the pharmacokinetics and drug delivery to the otic compartment (Piu and Bishop, 2019). Although there is a substantial medical need for a “tinnitus pill” (McFerran et al., 2019), many challenges hinder its development including tinnitus heterogeneity, insensitive outcome measures and the lack of objective measurements, impeding the translation from animal data to clinical application (Cederroth et al., 2018).

### 6.5. Other Interventions

#### 6.5.1. Non-invasive Neurostimulation

Non-invasive neuromodulation techniques, such as transcranial magnetic stimulation (TMS), transcranial electrical stimulation (tES), and neurofeedback (NFB), have also been used for the treatment of tinnitus. Traditionally, low-frequency repetitive TMS (rTMS) was introduced to reduce tinnitus-related hyperactivity of the auditory cortex (Langguth et al., 2003). Apart from several studies that investigated the effect of low-frequency rTMS in tinnitus (Schoisswohl et al., 2019), other trials examined the efficacy of continuous theta-burst stimulation (cTBS) (Plewnia et al., 2012; Schecklmann et al., 2016), high-frequency rTMS over the temporal cortex (Khedr et al., 2008, 2010), as well as multi site stimulation protocols such as an additional excitatory stimulation of the dorsolateral prefrontal cortex (DLPFC) (Lehner et al., 2013, 2016; Langguth et al., 2014; Formánek et al., 2018). Additionally, a combination of rTMS with specific medications (Kleinjung et al., 2009; Okamoto et al., 2010), relaxation techniques (Kreuzer et al., 2016), and peripheral muscle stimulation (Vielsmeier et al., 2018) have been investigated. However, there is still uncertainty regarding the efficacy of rTMS for the treatment of tinnitus, as indicated by several reviews with varying conclusions (Meng et al., 2011; Soleimani et al., 2016; Zenner et al., 2017; Londero et al., 2018), and two RCTs reporting conflicting results (Landgrebe et al., 2010; Folmer et al., 2015). Other than apparent differences in the investigated samples, potential reasons for inconsistent findings might be associated to gene polymorphisms related to neuroplasticity (Antal et al., 2010; Polania et al., 2018) or the intrinsic state prior stimulation (Silvanto and Pascual-Leone, 2008). For example, Weisz et al. (2012) demonstrated state-dependency effects of low-frequency rTMS applied over the auditory cortex. Another possible explanation could be the lack of clarity about appropriate stimulation parameters for the treatment of tinnitus (Schoisswohl et al., 2019). A promising approach to overcome this issue is provided by test sessions and the application of a reduced number of pulses. In test sessions, various stimulation frequencies or positions can be evaluated concerning brief tinnitus suppression to identify an efficient and individualized rTMS protocol (Schoisswohl et al., 2020). A pilot study already emphasized the utilization of personalized rTMS for the treatment of tinnitus (Kreuzer et al., 2017). Nevertheless, further systematic groundwork is needed to identify factors influencing the efficacy of rTMS in tinnitus.

In addition to TMS, since 2006 (Fregni et al., 2006), various forms of non-invasive tES have been explored as potential options for tinnitus management. tES is a neuromodulatory technique in which the weak current applied to the scalp is thought to modify neural processing (Bestmann and Walsh, 2017). The current can be constant, as is the case in transcranial direct current stimulation (tDCS), or alternating, as in transcranial random noise stimulation (tRNS) and transcranial alternating current stimulation (tACS). All three methods have been shown to increase cortical excitability in healthy subjects (Inukai et al., 2016). In tDCS, a small, constant current (1.0–2.0 mA) is applied to the scalp through two pad electrodes for 10–30 min. In tinnitus research, DLPFC has been targeted most often, followed by left temporoparietal area (LTA) (Kok et al., 2020). One challenge faced in tDCS research is the non-focal stimulation pattern created by the electrodes, which makes it challenging to target specific brain regions. Another technique called high-definition transcranial direct current stimulation (HD-tDCS) can overcome this as it results in more focal stimulation (Datta et al., 2009). Shekhawat et al. (2016) carried out a cross-over optimization study for HD-tDCS in tinnitus patients, comparing different stimulation intensities, duration, and locations. It was found that 77.78% of the participants were responsive to the treatment, with stimulation of 2.0 mA for 20 min to either DLPFC or LTA being the most effective stimulation paradigm. Jacquemin et al. (2018) compared tDCS of either LTA or DLPFC to HD-tDCS of DLPFC and found a clinically significant improvement in 32% of the participants regardless of stimulation position. Two systematic reviews with meta-analysis on the efficacy of tDCS for tinnitus have been carried out, showing a significant reduction in either tinnitus loudness (Song et al., 2012) or tinnitus-related distress (Wang et al., 2018) when compared to sham protocols. These reviews only included six and eight studies respectively and both concluded more RCTs are needed to draw firm conclusions. As in the case of TMS, there is a lack of replicability of results, which can be due to the difference in stimulation protocols and tinnitus heterogeneity. In tACS and tRNS, instead of a constant current, an alternating current is applied through the electrodes. To our knowledge, no systematic reviews have been published on the effect of these techniques on people with tinnitus. However, a recent narrative review of tES for tinnitus concluded that the effects of tACS, tRNS and tDCS are inconsistent and dependent on the method and montage used (Langguth, 2020).

Neurofeedback (NFB) is an individualized treatment approach, constructed on altered brain oscillations, which are recorded, analyzed and fed back to the participant in real-time. The feedback modality can be either visual, auditory, or tactile and is based on operant conditioning (Skinner, 1938). This trained reinforcement of brain activity patterns has been able to improve the psychological well-being, by reducing tinnitus distress and loudness in previous studies (Dohrmann et al., 2007a,b; Crocetti et al., 2011; Güntensperger et al., 2019). Despite the potential of NFB as a treatment for tinnitus, some aspects remain unresolved and require further investigation. First, the underlying mechanisms of NFB are not entirely comprehended, and discussions on its effects are ongoing (Fovet et al., 2017; Schabus, 2017; Witte et al., 2018). Further, only 15–30% of participants can modify their cortical brain activity (Alkoby et al., 2018), which is referred to as the inefficacy problem. This also raises the question of how to define a “responder” or “non-responder.” Given that only a subpopulation may benefit from NFB, the need for individualization of the treatment is evident. It can be addressed by including individually defined frequency bands (individual alpha peak) (Klimesch, 1999), accounting for heterogeneity in the analysis (Riha et al., 2020), and identifying predictors for a successful NFB training (Alkoby et al., 2018). The design of clinical studies (duration and frequency of training) and the nonexistence of a control or placebo group (Jensen et al., 2020) also need to be addressed. Detailed information on challenges in NFB can be found in (Gruzelier, 2014; Rogala et al., 2016; Hampson et al., 2019).

#### 6.5.2. Applications for Electronic Devices

Applications for electronic devices (apps) offer another potential management option for tinnitus. The number of apps in the field of otorhinolaryngology is increasing (Rak et al., 2019), as is the case for tinnitus apps (Sereda et al., 2019). Mehdi et al. (2020) provide an extensive review on tinnitus mobile apps, ranging from hearing protection, -testing, and -enhancement, to self-help (e.g., CBT and NFB), and relief (e.g., tinnitus masking). Unfortunately, the necessary medical quality criteria have not been developed concurrently, and there is a need for standard practice. Apart from app developers, highly qualified scientific and medical professionals have to participate in the development process and provide scientific evidence and integrity. Additionally, the question arises whether an app must be certified as a medical device, or if there should be a separate quality standard. For example, Stoyanov et al. (2015) proposed an objective and straightforward app-quality assessment tool of mobile health apps called MARS (Mobile App Rating Scale), which has already been applied in tinnitus apps (Mehdi et al., 2020). Another important consideration is that apps and integrated auxiliary and peripheral sensors, such as smartwatches, have the risk of unfiltered data storage and can lead to an infringement of personal interests.

#### 6.5.3. Combination of Interventions

As discussed for rTMS, various combinations of different approaches have been tried for tinnitus. Tinnitus retraining therapy (TRT) is another example of combining different therapeutic approaches (Jastreboff and Jastreboff, 2000; Bauer et al., 2017). TRT combines counseling and partial masking (either with sound generators or hearing aids) to modify pathological brain activity as well as promote habituation, and is widely used in clinical practice. However, there is mixed evidence on the efficacy of TRT (Grewal et al., 2014; Barozzi et al., 2017; Bauer et al., 2017; Zenner et al., 2017; Searchfield, 2020). In addition, different applications of simultaneous multimodal stimulation have shown promising results e.g., combination of sound and somatosensory stimulation (Marks et al., 2018; Conlon et al., 2020). Another example of combination of approaches is the ongoing UNITI clinical trial (Schlee et al., 2021), where different interventions, including CBT, structured counseling, sound therapy, and hearing aids will be investigated in a randomized controlled trial for their effects when delivered individually and in combination with one another.

In addition to these management options, research on numerous other potential interventions has been published. More exhaustive lists can be found in recent reviews from Fernandes et al. (2017) and Cima et al. (2019).

### 6.6. Challenges and Future Directions

One of the most critical factors when assessing the effectiveness of an intervention in a clinical trial is the selection and reporting of outcomes. A systematic review by Hall et al. (2016) aiming to identify and evaluate the outcome domains and instruments used in clinical trials of tinnitus treatments identified 35 different outcome domains and 78 different primary outcome instruments in clinical trials for tinnitus. However, not a single outcome was selected commonly across all studies. A need for a minimum outcome reporting standard for tinnitus is evident as it would ensure results of trials can be easily compared, contrasted, and combined when needed, enabling robust conclusions to be made about the effectiveness of any tinnitus interventions (Clarke and Williamson, 2016). The Core Outcome Measures in Tinnitus (COMiT) initiative aimed to establish an international standard for outcome measures in clinical trials of tinnitus. This was achieved by using a Delphi approach for three intervention types (sound-based, psychology-based and drug-based), leading to separate recommendations for each intervention type (Hall et al., 2018c, 2019a; Hibbert et al., 2020). This provided a critical starting point for the standardization of tinnitus research.

Besides the selection of outcome measures, there are many other important methodological aspects that need to be considered when designing a clinical trial, including randomization, blindness, and preregistration (Schulz et al., 2010; Nosek et al., 2018). Following good practice recommendations for clinical trials of any condition is essential to ensure study quality and reliability of any positive or negative findings. Equally important is to aim for large sample sizes based on power calculations, to account for tinnitus heterogeneity. In addition to this, especially in cases where large studies are impractical, selection of clearly characterized subphenotypes for inclusion is essential.

Another important topic to be addressed in future research is the role of precision medicine for tinnitus - an increasingly popular topic in mental health research (Fernandes et al., 2017). The term describes the effort to deliver a specific intervention depending on the patient particulars. These could include genetic and neuroimaging markers and psychoacoustic profiles, to name a few. As currently there is no individual treatment to cure tinnitus, and patients often respond differently to the clinically available interventions, a data-driven recommender system could assist clinicians by suggesting the most likely intervention to reduce the impact of tinnitus given a set of patient characteristics. The biggest challenge for such a system is identifying which characteristics may contribute to treatment response. Although personalized medicine has been proposed for tinnitus (Cederroth et al., 2019a), not many empirical studies have been conducted with such a goal. Simoes et al. (2019) showed that social demographics and tinnitus characteristics could partially explain the variance of self reported efficacy of 25 different interventions, suggesting that such a recommendation is theoretically feasible. In a follow up study, personality factors such as extraversion and neuroticism were implicated as significant factors on the variance of distress among patients who underwent clinical interventions (Simões et al., 2019). Many other factors, such as acoustic brain responses, neurophysiological markers from M/EEG, and behavioral data (e.g., coping strategies), that could have predictive power over treatment response, should be assessed by future research. Therefore, a comprehensive assessment of tinnitus (see Table 1) is recommended to both clinicians and researchers.

Congruent with the push for individualized medicine (Schork, 2015; Senn, 2018), Single-Case Experimental Designs (SCED) offer an alternative to RCTs. These designs rely on the continued observation of a unit (e.g., an individual, group, hospital) under different conditions of at least one manipulated variable for a predetermined period of time. As such, SCEDs are capable of establishing robust causal relationships and may provide high levels of evidence (for an overview and historical context see Vlaeyen et al., 2020). While the strengths of such designs are reflected in the growing interest (Hammond and Gast, 2010) and the development of guidelines (Tate et al., 2016), this methodology is still under-explored in the tinnitus field. The idiosyncratic nature of tinnitus provides a rich soil where SCED may thrive by providing robust and individualized baselines to which treatment effects are compared. Protocols may be more easily replicated than RCTs and allow for SCEDs to be carried out in different clinics across countries. Advances in methodology (e.g., Bulté and Onghena, 2013) permit for such research to contribute to meta-analysis and big data models, as several data points are collected per participant. As such, SCED offers a viable solution for the development of research focusing on the intraindividual variability of tinnitus patients.

## 7. Future Research Directions

In this section, four broad research directions are further discussed based on recommendations from previous sections. A summary of these can be found in Tables 2, 3.

**Table 2:** Overview of challenges in tinnitus research and proposed future direction towards a cure for tinnitus.

**Challenges**	**Proposed directions**
**Tinnitus Heterogeneity**	
Lack of understanding of tinnitus heterogeneity and no clearly defined subtypes	Systematic reviews of studies investigating tinnitus subphenotypes Standardization in tinnitus assessment and international collaborations to create large tinnitus-specific databases Exploratory analyses investigating multiple dimension of tinnitus heterogeneity in large samples to identify important phenotypes
**Epidemiology, Risk Factors & Prevention**	
Varying prevalence estimates and associations between tinnitus and other factors	Standardization of tinnitus definition Systematic reviews and retrospective analysis of existing databases Prospective analytical observational studies from nationally representative populations
**Tinnitus Mechanisms**	
Unknown underlying pathophysiology and lack of replicable functional, structural and neurochemical neural markers and genetic markers of tinnitus	Systematic reviews on methods and results of previous studies Standardization of data acquisition and analysis methods Large studies investigating suspected theories Replication of existing studies Selection of clear phenotypes for inclusion
**Tinnitus Assessment**	
Lack of standardization in tinnitus assessment (what and how)	Systematic reviews to identify the most useful and reliable measures Collaboration among research groups and consensus projects to standardize tinnitus assessment
**Treatments**	
Low evidence from clinical trials	Large randomized controlled clinical trials based on sample power calculations Pre-registration Consensus on outcome measures Treatment predictors and personalized medicine Single-case design methodology Selection of clear phenotypes for inclusion

**Table 3:** Common objectives identified across different domains of tinnitus research as summarized in Table 2.

**Summary of future directions for tinnitus research**
International and interdisciplinary collaborations
Reviews of existing knowledge
Standardization in research methods
Big data

First, establishing additional interdisciplinary, multicenter collaborations was identified as an important research direction. Some examples such as TIGER, ESIT, TIN-ACT, and UNITI underscore the benefit of such interdisciplinary collaborations to advance the understanding of tinnitus heterogeneity, pathophysiology, and genetic markers, and to develop well-designed, multicenter, randomized clinical trials. Additionally, we recommend researchers to engage with non-profit, patient-oriented organizations such as the British Tinnitus Association (BTA), the American Tinnitus Association (ATA) and TinnitusHub. Such collaborations leading to patient and public involvement (PPI) are highly regarded as a way to improve research quality (Brett et al., 2014), but remain uncommon in the tinnitus field. An update of the almost 10-year old James Lind Alliance priority research for tinnitus (Hall et al., 2013) exemplifies the type of PPI that would greatly benefit both researchers and patients.

Second, a lack of reviews of existing knowledge was indicated as an impediment to progress in tinnitus research. For example, a need for further Cochrane reviews for specific tinnitus interventions has recently been proposed (Sereda et al., 2020), since reviews for some commonly prescribed interventions are missing or are almost a decade old (Baldo et al., 2012). Additionally, systematic reviews would significantly contribute to selecting among the different available methods for assessing an individual with tinnitus and inform decisions for standardizing tinnitus assessment (Shabbir et al., 2021). The lack of summarized data available in biobanks is another example that highlights the need for more systematic reviews. As previously discussed, small sample sizes was identified as a hindrance to a greater understanding of tinnitus heterogeneity and pathophysiology. Although biobanks could be used to increase sample size, few studies have done so in the tinnitus field. A review could aid researchers aiming to use “Big Data” by summarizing which biobanks have information on tinnitus patients, what type of data is available (e.g., neuroimaging, biomarkers, audiological examination, assessment of comorbidities), and the relevant published literature.

Third, standardizing research methods, such as tinnitus assessment and data analysis protocols, is paramount. Although consensus-based initiatives such as TRI and COMiT offer guidelines for tinnitus assessment and outcome measures, these guidelines are not consistently followed. As a result, comparison between diverging results is problematic. For example, it is common for clinical trials to differ in primary/secondary outcomes, quantity and duration of follow-ups, reports of inclusion/exclusion criteria, and reported demographics information (Hall et al., 2018c). Any of those factors could explain why seemingly identical studies lead to different outcomes, thus obfuscating attempts to better understand heterogeneity. Another example is the inconsistencies in data acquisition and analysis methods in neuroimaging studies, a common problem in many research areas (Botvinik-Nezer et al., 2020), hindering comparison of results across studies.

Lastly, the lack of large-sampled studies has been identified as a major deterrent to progress in the field. Here we present some suggestions to incorporate “Big Data” in tinnitus research. First, biobanks are still rarely used in the tinnitus field despite their large multimodal data. As mentioned previously, no reviews have summarized the tinnitus-relevant contents of biobanks nor which studies have been done with this type of data. Some biobanks, such as the UK Biobank, have tens of thousands of tinnitus patients in their records, including neuroimaging data, biological material, genetic material, and questionnaires. Nonetheless, only a few studies have been published with data from the UK Biobank (Bycroft et al., 2018). Despite being a promising alternative to tackle issues related to unknown pathophysiology, genetics, biomarkers and heterogeneity, the challenging nature of data analysis from Biobanks reinforces the aforementioned need for interdisciplinary collaborations and standardization in research methods. Second, apps could be used not only to deliver treatments (see treatments section), but also to provide ecological insights about tinnitus that would be difficult to access in traditional data collection settings. For instance, a prospective study using app data showed that tinnitus loudness usually fluctuates throughout the day, even when patients initially reported otherwise (Pryss et al., 2019). With mobile apps, thousands of observations from users can be collected, and patterns of variation in tinnitus loudness and distress could be used to identify potential tinnitus subtypes. For a review on this topic, see (Mehdi et al., 2019). Similarly, online recruitment databases could be used to increase sample sizes and, in turn, capture aspects of tinnitus heterogeneity different from clinical samples. A previous study has shown that the demographics of tinnitus patients from clinics are different from patients recruited through online platforms (Probst et al., 2017), indicating that sample bias could hamper the understanding of tinnitus heterogeneity.

In summary, shedding light to a complex phenomenon like tinnitus requires interdisciplinary expertise. The review of research challenges from multiple tinnitus domains led to the conclusion that coordinated efforts among tinnitus researchers is essential. These should focus on synthesizing and reviewing existing knowledge, identifying research gaps, standardizing research methodologies, and creating large tinnitus-specific databases that would allow in-depth exploration of tinnitus heterogeneity.

## Author Contributions

Coordination: ED, EG, JPS, and MS. Writing-Tinnitus heterogeneity section: EG (writing coordinator), JPS, CC, and FE. Writing-Epidemiology, Risk Factors & Prevention section: JPS (writing coordinator), RB. Writing-Tinnitus Mechanisms section: EG (writing coordinator), ALR, LJ, NL, and PM (hearing deprivation and brain plasticity). ALR, PM, SS (neuroimaging), NT, and SA (tinnitus genetics). Writing-Tinnitus Assessment section: MS (writing coordinator), EG, MS, RB (case history assessment), CC, ED, EG, and JLS (audiological assessment). Writing-Treatment Development section: ED (writing coordinator, pharmacology-based interventions), ML (psychology-based interventions), MJ, SS (sound generators), MJ, JLS (hearing aids), KA, NL (cochlear implants), SS (rTMS), TK (tES), and CR (neurofeedback, mobile applications). Writing-Future Research Directions section: JPS (writing coordinator), AD, AS, EG, JLS, MM, and RB. Conceptualization, writing-tables, review, and editing: all authors. All authors have read and agreed to the final version of the manuscript.

## Conflict of Interest

KA is employed by Cochlear Company. MJ is employed by WS Audiology. CC is employed by Oticon A/S. These relationships have not influenced the work reported in this paper. The remaining authors declare that the research was conducted in the absence of any commercial or financial relationships that could be construed as a potential conflict of interest.
