# Corrigendum: Multidisciplinary Tinnitus Research: Challenges and Future Directions From the Perspective of Early Stage Researchers

**DOI:** 10.3389/fnagi.2021.730758

**Published:** 2021-08-09

**Authors:** Jorge Piano Simoes, Elza Daoud, Maryam Shabbir, Sana Amanat, Kelly Assouly, Roshni Biswas, Chiara Casolani, Albi Dode, Falco Enzler, Laure Jacquemin, Mie Joergensen, Tori Kok, Nuwan Liyanage, Matheus Lourenco, Punitkumar Makani, Muntazir Mehdi, Anissa L. Ramadhani, Constanze Riha, Jose Lopez Santacruz, Axel Schiller, Stefan Schoisswohl, Natalia Trpchevska, Eleni Genitsaridi

**Affiliations:** ^1^Department of Psychiatry and Psychotherapy, University of Regensburg, Regensburg, Germany; ^2^Centre National de la Recherche Scientifique, Aix-Marseille University, Marseille, France; ^3^Hearing Sciences, Mental Health and Clinical Neurosciences, School of Medicine, University of Nottingham, Nottingham, United Kingdom; ^4^Otology & Neurotology Group CTS 495, Department of Genomic Medicine, GENYO - Centre for Genomics and Oncological Research Pfizer/University of Granada/Junta de Andalucía, PTS, Granada, Spain; ^5^Department of Otorhinolaryngology and Head & Neck Surgery, University Medical Center Utrecht, Utrecht, Netherlands; ^6^Department of Clinical and Experimental Neuroscience, University Medical Center Utrecht Brain Center, Utrecht University, Utrecht, Netherlands; ^7^Cochlear Technology Centre, Mechelen, Belgium; ^8^Laboratory of Lifestyle Epidemiology, Department of Environmental Health Sciences, Istituto di Ricerche Farmacologiche Mario Negri IRCCS, Milan, Italy; ^9^Hearing Systems, Department of Health Technology, Technical University of Denmark, Lyngby, Denmark; ^10^Oticon A/S, Smoerum, Denmark; ^11^Interacoustics Research Unit, Lyngby, Denmark; ^12^Institute of Databases and Information Systems, Ulm University, Ulm, Germany; ^13^Department of Otorhinolaryngology Head and Neck Surgery, Antwerp University Hospital, Edegem, Belgium; ^14^Department of Translational Neurosciences, Faculty of Medicine and Health Sciences, Antwerp University, Wilrijk, Belgium; ^15^WS Audiology, Lynge, Denmark; ^16^Ear Institute, University College London, London, United Kingdom; ^17^University of Zurich, Zurich, Switzerland; ^18^Department of Otorhinolaryngology, Head and Neck Surgery, University Hospital Zurich, Zurich, Switzerland; ^19^Experimental Health Psychology, Faculty of Psychology and Neuroscience, Maastricht University, Maastricht, Netherlands; ^20^Health Psychology Research Group, Faculty of Psychology and Educational Sciences, University of Leuven, Leuven, Belgium; ^21^Department of Otorhinolaryngology, Head and Neck Surgery, University of Groningen, University Medical Center Groningen, Groningen, Netherlands; ^22^Graduate School of Medical Sciences (Research School of Behavioral and Cognitive Neurosciences), University of Groningen, Groningen, Netherlands; ^23^Institute of Distributed Systems, Ulm University, Ulm, Germany; ^24^Radiological Sciences, Mental Health and Clinical Neurosciences, School of Medicine, University of Nottingham, Nottingham, United Kingdom; ^25^Sir Peter Mansfield Imaging Centre, School of Medicine, University of Nottingham, Nottingham, United Kingdom; ^26^Chair of Neuropsychology, Department of Psychology, University of Zurich, Zurich, Switzerland; ^27^Department of Physiology and Pharmacology, Experimental Audiology Laboratory, Karolinska Institutet, Stockholm, Sweden; ^28^Nottingham Biomedical Research Centre, National Institute for Health Research, Nottingham, United Kingdom

**Keywords:** tinnitus, review, heterogeneity, standardization, interdisciplinary collaborations, big data, treatment development

In the original article, there was an error. For the sentence “NMDA receptor antagonists (AM-101) have been discontinued in phase III for not meeting endpoints (van de Heyning et al., 2014)” there was a typographical error (phase III should have been phase II). In addition, it was brought to our attention that clinical trials for AM-101 are ongoing.

A correction has been made to section 6. Treatment Development, Subsection 6.4. Pharmacology-Based Interventions, paragraph 1. The corrected paragraph is below.

A wide variety of therapeutic drugs have been used to relieve tinnitus (Elgoyhen and Langguth, 2010). For acute tinnitus, a dose-dependent reduction in tinnitus intensity was observed with intravenous lidocaine (Trellakis et al., 2006). However, its use is controversial due to its short-lasting response, its potentially life threatening arrhythmogenic side effects, and the low bioavailability of its oral form (Israel et al., 1982; Trellakis et al., 2007; Gil-Gouveia and Goadsby, 2009). A potential goal of pharmacologic tinnitus research could be to identify the mechanism by which lidocaine interferes with tinnitus and mimic this effect using a drug with better tolerance that can be orally administered. For chronic tinnitus, the off-label use of medicines like betahistine (Hall et al., 2018d), anticonvulsants (Hoekstra et al., 2011), and glutamate receptor antagonists have shown little or no effect in clinical trials. Prescription of antidepressants and benzodiazepines is limited to tinnitus-associated comorbidities such as depression, insomnia and anxiety (Langguth et al., 2019). Moreover, three clinical research programs, in the last few years, were discontinued in phase II and III. AMPA antagonist selurampanel (BGG492) has not resulted in a new compound (Cederroth et al., 2018). NMDA receptor antagonists (AM-101) did not meet the primary endpoint of improving minimum masking level in acute tinnitus in a phase II clinical trial but showed improvement for tinnitus loudness, annoyance, sleep difficulties, and tinnitus impact in patients with tinnitus after noise trauma or otitis media (van de Heyning et al., 2014). Many other treatments decreasing tinnitus percept or targeting central auditory processing pathways are at a preclinical phase (Schilder et al., 2019). The modulator of voltage-gated potassium channels (Kv3.1) (AUT00063) was not effective in alleviating tinnitus symptoms (Hall et al., 2019b).

The authors apologize for this error and state that this does not change the scientific conclusions of the article in any way. The original article has been updated.

## Publisher's Note

All claims expressed in this article are solely those of the authors and do not necessarily represent those of their affiliated organizations, or those of the publisher, the editors and the reviewers. Any product that may be evaluated in this article, or claim that may be made by its manufacturer, is not guaranteed or endorsed by the publisher.
